# Supplementary material for: Linking influenza virus evolution within and between human hosts
Source: Virus Evol. 2020 Feb 17;6(1):veaa010. doi: 10.1093/ve/veaa010 (PMC7025719; doi:10.1093/ve/veaa010)
Supplement: veaa010_Supplementary_Data [file veaa010_supplementary_data.zip › FigureS3-SampleExclusions-caption.pdf]

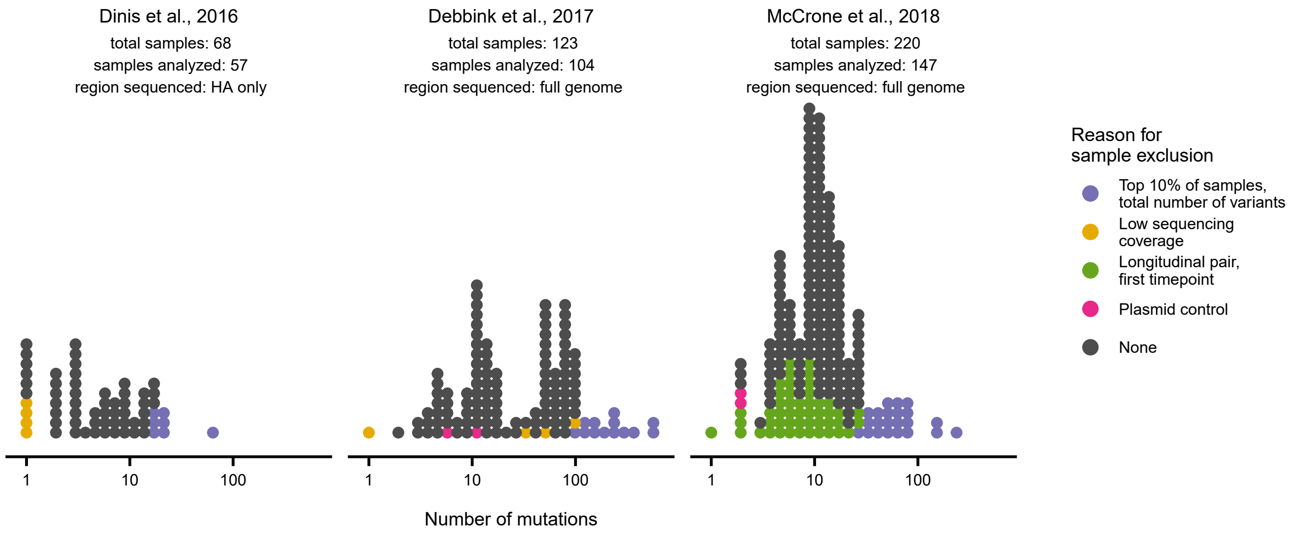

Figure S3. Samples excluded from downstream analyses. Each point represents the number of mutations identified above a frequency of 0.5% in a patient sample. Samples colored in grey were included in subsequent analyses. Samples in purple ranked in the top 10% of samples sequenced in that study based on the number of within-host mutations. Samples in yellow had incomplete sequencing coverage, meaning that fewer than 80% of sites in at least one gene were sequenced to 400x coverage. Samples in green were the first members of longitudinal pairs of samples obtained from the same patient infection; the first sample in each longitudinal pair was excluded to remove potential correlations in viral diversity between samples from a single patient. Samples in pink were plasmid controls that do not represent clinical viral populations.
