# Supplementary material for: Linking influenza virus evolution within and between human hosts
Source: Virus Evol. 2020 Feb 17;6(1):veaa010. doi: 10.1093/ve/veaa010 (PMC7025719; doi:10.1093/ve/veaa010)
Supplement: veaa010_Supplementary_Data [file veaa010_supplementary_data.zip › FigureS4-AcuteRates-ByStudy-0.005-caption.pdf]

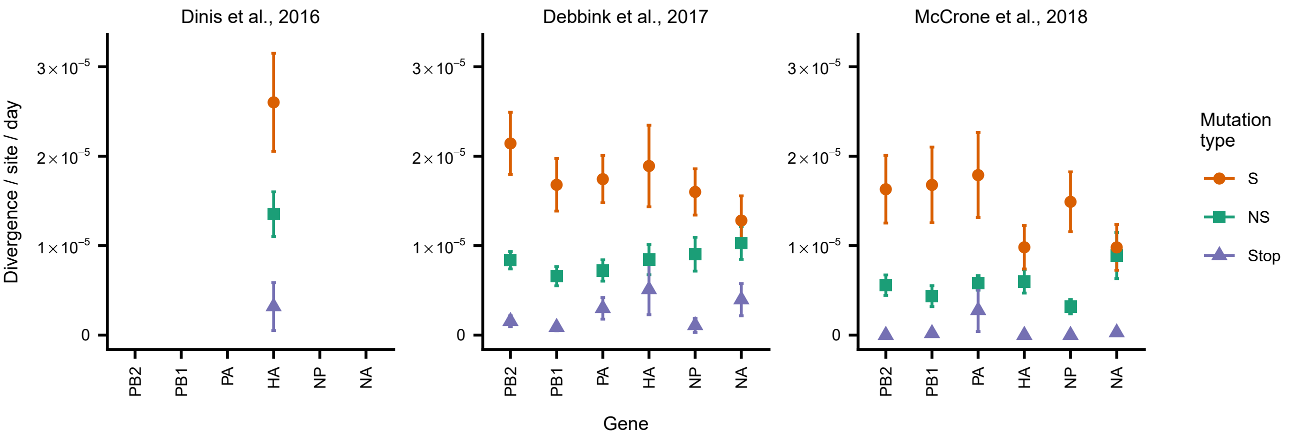

Figure S4. Estimates of within-host evolutionary rates are broadly consistent across cohorts. Shown are the mean and standard error of within-host evolutionary rates calculated as described in **Figure 3** for viral samples in each published dataset. Note that (Dinis et al., 2016) sequenced only the HA gene.
