# Supplementary material for: Linking influenza virus evolution within and between human hosts
Source: Virus Evol. 2020 Feb 17;6(1):veaa010. doi: 10.1093/ve/veaa010 (PMC7025719; doi:10.1093/ve/veaa010)
Supplement: veaa010_Supplementary_Data [file veaa010_supplementary_data.zip › FigureS5-AcuteRates-CompareRegression-0.005-caption.pdf]

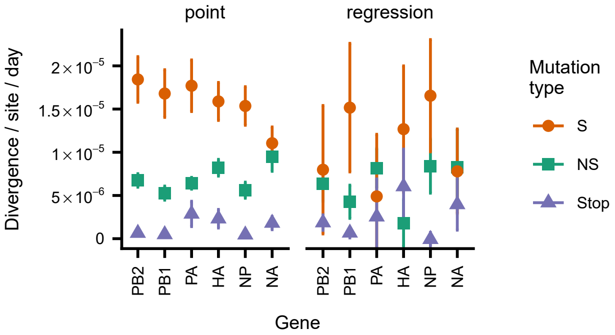

Figure S5. Within-host evolutionary rates show qualitatively similar trends when calculated using different methods. Evolutionary rates estimated using the point method are described and shown in **Figure 3**. Evolutionary rates were estimated using the regression method by calculating the total divergence of each within-host viral population, normalizing to the number of available sites, and then performing linear regression of per-site viral divergence by the time elapsed since each infection began. Rates estimated using the regression method do not include samples from the (Dinis et al., 2016) study because metadata on the timing of sample collection was only available for samples in aggregate for this dataset rather than the samples individually. (Rates estimated using the point method use aggregate metadata on the timing of sample collection.)
