# Supplementary material for: Linking influenza virus evolution within and between human hosts
Source: Virus Evol. 2020 Feb 17;6(1):veaa010. doi: 10.1093/ve/veaa010 (PMC7025719; doi:10.1093/ve/veaa010)
Supplement: veaa010_Supplementary_Data [file veaa010_supplementary_data.zip › FigureS6-ChronicRates-caption.pdf]

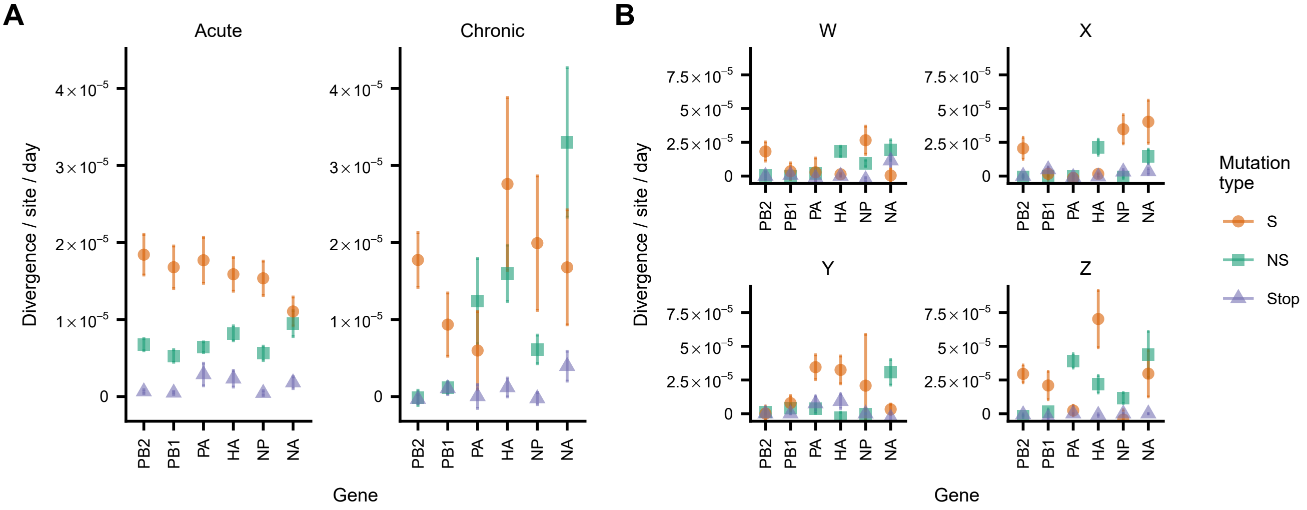

Figure S6. Within-host evolutionary rates in chronic influenza infections. A) Within-host evolutionary rates in acute and chronic infections. Evolutionary rates in acute infections were calculated as in **Figure 3**. Evolutionary rates in chronic infections were estimated separately for each of four patients from previously sequenced longitudinal viral samples (Xue et al., 2017) by calculating the total divergence of viral populations at each time point, normalizing to the number of available sites, and performing linear regression of per-site viral divergence by time since the infection began (see Materials and methods). Shown here are the mean and standard error of the evolutionary rates estimated for each patient. B) Within-host evolutionary rates plotted separately for each patient. Shown are the mean and standard error of evolutionary rates estimated as described above through linear regression. Patients are named as in (Xue et al., 2017).
