# Supplementary material for: Linking influenza virus evolution within and between human hosts
Source: Virus Evol. 2020 Feb 17;6(1):veaa010. doi: 10.1093/ve/veaa010 (PMC7025719; doi:10.1093/ve/veaa010)
Supplement: veaa010_Supplementary_Data [file veaa010_supplementary_data.zip › FigureS7-GlobalSequenceDistances-caption.pdf]

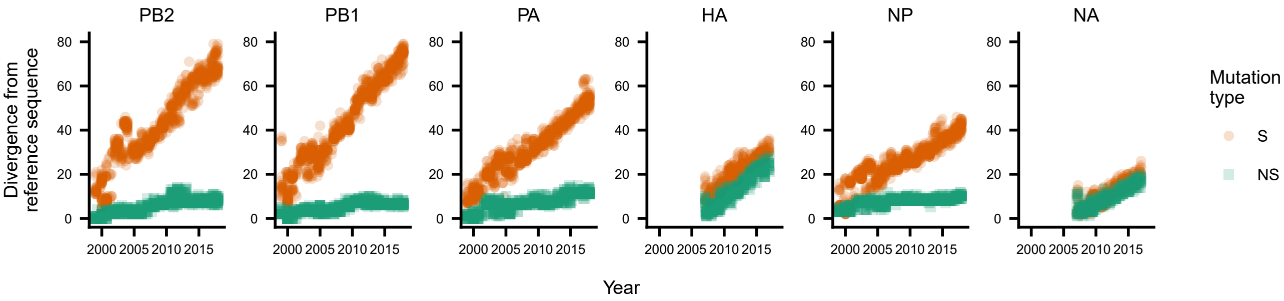

Figure S7. Estimate of global evolutionary rates using a molecular-clock method. Synonymous and nonsynonymous sequence divergences from a reference sequence are shown for randomly sampled sequences from the GISAID database (Bogner et al., 2006). For the PB2, PB1, PA, and NP genes, sequences from 1999-2017 were analyzed relative to a A/Moscow/10/1999 reference sequence. For the HA and NA genes, which evolve rapidly and can quickly saturate available sites of mutation, sequences from 2007-2017 were analyzed relative to a A/Brisbane/10/2007 reference sequence. Outlier sequences, which likely result from mis-annotations, were removed prior to performing this analysis as described in Materials and methods.
