# Supplementary material for: Linking influenza virus evolution within and between human hosts
Source: Virus Evol. 2020 Feb 17;6(1):veaa010. doi: 10.1093/ve/veaa010 (PMC7025719; doi:10.1093/ve/veaa010)
Supplement: veaa010_Supplementary_Data [file veaa010_supplementary_data.zip › FigureS1-MetadataDPIs-caption.pdf]

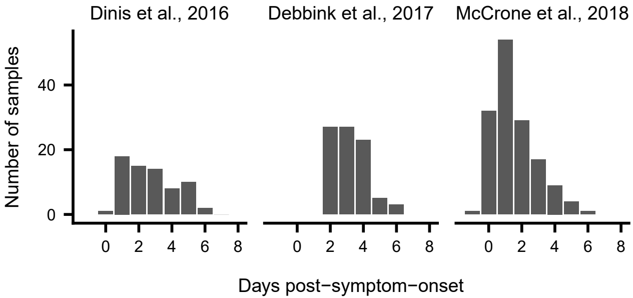

Figure S1. Distribution of days post-symptom-onset on which viral samples were collected. Note that symptoms typically emerge about 2 days after viral infection begins (Baccam et al., 2006; Carrat et al., 2008). Samples from the (Debbink et al., 2017) and (McCrone et al., 2018) studies that were excluded from analysis (see Materials and methods) were omitted from these distributions. All samples from the (Dinis et al., 2016) study are shown here, even though some of these samples were excluded from subsequent analyses, because metadata on the timing of sample collection was only available for all H3N2 samples in aggregate rather than for each sample individually.
