# Supplementary material for: Linking influenza virus evolution within and between human hosts
Source: Virus Evol. 2020 Feb 17;6(1):veaa010. doi: 10.1093/ve/veaa010 (PMC7025719; doi:10.1093/ve/veaa010)
Supplement: veaa010_Supplementary_Data [file veaa010_supplementary_data.zip › FigureS2-AcuteRates-ByMinFreq-caption.pdf]

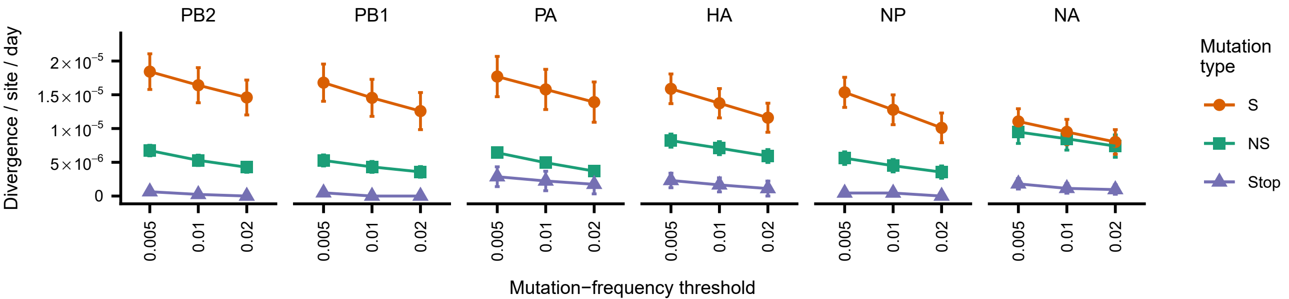


**Figure S2**. Estimates of within-host evolutionary rates are robust to variant-frequency thresholds. Shown are the mean and standard error of within-host evolutionary rates calculated as described in **Figure 3** for commonly used variant-frequency thresholds.
